# CX Chemokine Receptor 7 Contributes to Survival of *KRAS*-Mutant Non-Small Cell Lung Cancer upon Loss of Epidermal Growth Factor Receptor

**DOI:** 10.3390/cancers11040455

**Published:** 2019-03-30

**Authors:** Bin Liu, Shanshan Song, Rita Setroikromo, Siwei Chen, Wenteng Hu, Deng Chen, Anthonie J. van der Wekken, Barbro N. Melgert, Wim Timens, Anke van den Berg, Ali Saber, Hidde J. Haisma

**Affiliations:** 1 Groningen Research Institute of Pharmacy, Department of Chemical and Pharmaceutical Biology, University of Groningen, 9713 AV Groningen, The Netherlands; bin.liu@rug.nl (B.L.); s.song@rug.nl (S.S.); r.setroikromo@rug.nl (R.S.); cherry.chen@rug.nl (S.C.); d.chen.5@student.rug.nl (D.C.); a.saber814@gmail.com (A.S.); 2 Toxicology and Targeting Groningen Research Institute for Pharmacy, Department of Pharmacokinetics, University of Groningen, 9713 AV Groningen, The Netherlands; b.n.melgert@rug.nl; 3 Department of Thoracic Surgery, The First Hospital of Lanzhou University, Lanzhou 730000, China; huwenteng08@163.com; 4 Department of Pulmonary Diseases, University Medical Centre Groningen, University of Groningen, 9713 GZ Groningen, The Netherlands; a.j.van.der.wekken@umcg.nl; 5 GRIAC- Groningen Research Institute for Asthma and COPD, University Medical Center Groningen, University of Groningen, 9713 GZ Groningen, The Netherlands; 6 Department of Pathology and Medical Biology, University Medical Center Groningen, University of Groningen, 9713 GZ Groningen, The Netherlands; w.timens@umcg.nl (W.T.); a.van.den.berg01@umcg.nl (A.v.d.B.)

**Keywords:** epidermal growth factor receptor (EGFR), CXCR7, KRAS, lung cancer, targeted therapy, gene editing

## Abstract

KRAS-driven non-small cell lung cancer (NSCLC) patients have no effective targeted treatment. In this study, we aimed to investigate targeting epidermal growth factor receptor (*EGFR*) as a therapeutic approach in KRAS-driven lung cancer cells. We show that ablation of *EGFR* significantly suppressed tumor growth in KRAS-dependent cells and induced significantly higher expression of CX chemokine receptor 7 (CXCR7) and activation of MAPK (ERK1/2). Conversely, rescue of EGFR led to CXCR7 downregulation in *EGFR*^−/−^ cells. Dual EGFR and CXCR7 inhibition led to substantial reduction of MAPK (pERK) and synergistic inhibition of cell growth. Analysis of two additional *EGFR* knockout NSCLC cell lines using CRISPR/Cas9 revealed genotype dependency of CXCR7 expression. In addition, treatment of different cells with gefitinib increased CXCR7 expression in *EGFR^wt^* but decreased it in *EGFR^mut^* cells. CXCR7 protein expression was detected in all NSCLC patient samples, with higher levels in adenocarcinoma as compared to squamous cell lung carcinoma and healthy control cases. In conclusion, EGFR and CXCR7 have a crucial interaction in NSCLC, and dual inhibition may be a potential therapeutic option for NSCLC patients.

## 1. Introduction

Lung cancer is the leading cause of cancer related death worldwide [1]. Non-small cell lung cancer (NSCLC) accounts for approximately 85% of lung cancers [2]. Different options for the treatment of patients with NSCLC are available, including surgery, radiotherapy, chemotherapy, immunotherapy, and tyrosine kinase inhibitors (TKIs). Gefitinib, erlotinib, and afatinib are the most commonly used TKIs directed against mutant epidermal growth factor receptor (EGFR) [3]. Despite high response rates in *EGFR*-mutant NSCLC patients, the majority of the patients cannot benefit from EGFR-TKIs, as the frequency of the activating mutations is about 10% in the non-Asian population [4]. The objective response rate in patients with wild-type *EGFR* treated with TKIs (7.2%) is significantly lower as compared to the chemotherapy (16.8%) group [5].

*KRAS* is mutated in 20–30% of NSCLC cases and is involved in the regulation of cell proliferation. Mutations in *KRAS* mostly occur in codon 12 and 13 and are associated with a worse prognosis. Patients with mutant *KRAS* have worse response to EGFR-TKIs, radiotherapy, and adjuvant chemotherapy [6]. Therapeutic approaches targeting KRAS are still limited with low clinical efficacy. Therefore, *KRAS*-mutant tumors are considered as “undruggable.”

There is ample evidence that resistance to the EGFR-TKIs can be related to reactivation of the MAPK/ERK signaling pathway [7]. The reactivation of MAPK/ERK signaling limits the response to EGFR-TKIs and leads to resistance [8,9]. Single targeted therapy for MAPK/ERK associated pathways, such as anti-BRAF, -MEK and -KRAS, is inefficient because tumor cells can acquire resistance within a short time by activating alternative pathways [10,11]. Pre-clinical and clinical studies using combination therapy have shown more promising results in TKI-resistant tumors with alterations in MAPK/ERK pathways, for instance, the dual inhibition of KRAS/FAS, KEAP1/NRF2, BRAF/MEK, and BRAF/RTK [7,10,11,12,13].

Crosstalk between G protein-coupled receptor (GPCRs) and EGFR contributes to tumor cell progression [14]. CX chemokine receptor 7 (CXCR7) is a new member of GPCRs [15], which for a long time had been considered as a receptor for vasoactive intestinal peptide and as a decoy receptor [16,17]. Recently, CXCR7 has been classified as a novel receptor for CX chemokine ligand (CXCL) 12 [18], CXCL11, human macrophage migration inhibitory factor (MIF) [19], and Dickkopf-3 [20]. CXCR7 facilitates tumor development and progression [21,22]. CXCR7 is also involved in the formation of metastasis in lung cancer patients/mouse models [21,23,24]. CXCR7 may interact with EGFR and promote MAPK signaling and tumor cell progression [25,26,27,28]. Interestingly, one study shows that secreted MIF binds to EGFR [29] and inhibits its activation. Another study showed that MIF binds to CXCR7 and promotes ERK signaling [19]. In addition, co-localization of CXCR7 with EGFR leads to EGFR phosphorylation [28]. Despite these studies, the mechanisms behind CXCR7-EGFR crosstalk and how CXCR7 behaves during EGFR targeted treatment are not clear.

In this study, we aimed to explore *EGFR* knockout as a therapeutic option in *EGFR* wild-type and *KRAS* mutated lung cancer cells. To the best of our knowledge, this is the first study showing that wild-type *EGFR* plays a significant role in growth of KRAS-dependent cancer cells. We identified overexpression of CXCR7 as a bypass mechanism in *EGFR*^−/−^ cells by promoting MAPK signaling. Both EGFR and CXCR7 inhibition showed synergetic suppression of cancer cell growth. Furthermore, we revealed that CXCR7 was increased in *EGFR^wt^* but decreased in *EGFR^mut^* cell lines after treatment with gefitinib. We also show that CXCR7 expression is higher in adenocarcinoma (ADC) than squamous cell lung carcinoma (SQCC) in patients with NSCLC and healthy lung tissue. Hence, dual inhibition of EGFR and CXCR7 might be a potential treatment strategy for NSCLC.

## 2. Materials and Methods

### 2.1. Cell Culture

A549 (wild-type *EGFR*, mutant *KRAS*) was purchased from ATCC. H1650 (mutant *EGFR*, wild-type *KRAS*) and H1299 (wild-type *EGFR*, wild-type *KRAS*) cell lines were kindly provided by Dr. Klaas Kok (Department of Genetics, University Medical Center Groningen, Groningen, The Netherlands). The HCC827 (mutant *EGFR*, wild-type *KRAS*) cell line was a gift from Dr. Martin Pool (Department of Medical Oncology, University Medical Center Groningen, Groningen, The Netherlands). The cells cultured either in DMEM (A549) or RPMI-1640 (H1650, H1299, and HCC827) containing 1% penicillin/streptomycin supplemented with 10% fetal bovine serum (FBS) (Costar Europe, Badhoevedorp, The Netherlands) at 37 °C with 5% CO_2_. Cells were stimulated with 10 ng/mL epidermal growth factor (EGF) (R & D Systems, Minneapolis, MN, USA) for 15 min or with recombinant human SDF-1α (CXCL12) (300-28A) (PeproTech, Rocky Hill, CT, USA) for 3 min, where indicated.

### 2.2. Anti-Tumor Reagents

Cetuximab was purchased from Merck (Dietikon, Switzerland). Gefitinib was purchased from Sigma-Aldrich (Zwijndrecht, The Netherlands); Y27632 was purchased from Tocris Bioscience (Bristol, UK). Sunitinib, selumetinib, and vemurafenib were purchased from LC Laboratories (Woburn, MA, USA). C646 was purchased from Axon Medchem (Groningen, the Netherlands). SAHA and entinostat were purchased from Selleckchem (Munich, Germany). Cis-Diamineplatinum (II) dichloride and staurosporine were purchased from Sigma (Aldrich, Nederland). Doxrubicin was purchased from Teva Pharmaceuticals. VI83, which inhibits PDGFRß, was a kind gift from Vichem Laboratories (Budapest, Hungary). CXCR7 inhibitor was a kind gift from Professor Rob Leurs and Professor Martine J. Smit (Vrije Universiteit Amsterdam, Amsterdam, The Netherlands). All drugs were diluted and aliquoted in dimethyl sulfoxide (DMSO), stored at −20 °C.

### 2.3. Transfection and Establishment of EGFR^−/−^ Cell Lines

A pool of two CRISPR/Cas9 *EGFR* knockout (KO) plasmids (Santa Cruz Biotechnology, Dallas, TX, USA), each encoding the Cas9 nuclease and a 20-nucleotide guide RNA (gRNA) targeting exons 2 and 3 of the *EGFR*, were used to establish *EGFR*^−/−^ lung cancer cell lines (Table S1). For transfection, 3 × 10^5^ cells were seeded in a single well of a 6-well plate. The next day, cells were transfected with CRISPR/Cas9 plasmids pool using Lipofectamine 3000 (Invitrogen, Carlsbad, USA) according to the manufacturer’s instructions with 3 μg of CRISPR/Cas9 plasmids. These plasmids contain GFP (Santa Cruz Biotechnology, Dallas, TX, USA) and puromycin resistance genes. Puromycin was used to select cells with successful uptake of the plasmid. Culture medium was changed one day after transfection, followed by puromycin selection with 2 μg/mL for the next three days. Single cells were seeded into 96-well plates and after three weeks of culture single cell wells were tested for *EGFR* knockout by Sanger sequencing, Western blot, and fluorescence-activated cell sorting (FACS) analysis. We could not establish HCC827 *EGFR*^−/−^ cells, which is probably due to the strong dependence of these cells to EGFR signaling [30,31].

### 2.4. Western Blot

Cells were lysed using ELB-softer (150 mM NaCl, 50 mM Hepes pH = 7.5, 5 mM EDTA, 0.1% NP-40) with Protease Inhibitor Cocktail (Thermo Fisher Scientific, Waltham, MA, USA), and PhosSTOP Phosphatase Inhibitor Cocktail (Roche, Mannheim, Germany). Protein concentrations were measured using a Pierce BCA Protein Assay Kit (Thermo Fisher Scientific, Waltham, MA, USA) according to the manufacturer’s protocol. Twenty micrograms of each sample were separated by pre-cast 5–15% SDS-PAGE (Bio-Rad, Hercules, CA, USA) and transferred to a polyvinylidene difluoride (PVDF) membrane. The membrane was blocked with 5% skimmed milk in PBST for 1 h at room temperature (RT) and incubated overnight at 4 °C with the following primary antibodies—MAPK (Erk) Antibody (#9102, 1:1000), Akt Antibody (#9272, 1:1000), Phospho-EGF Receptor (Tyr1068) (#2234, 1:1000), p(Thr308)-Akt (#9275, 1:1000), Phospho-Akt (Ser473) (#9271, 1:1000), Phospho-MAPK (pERK) (#9101, 1:1000), and β-Actin (#4967, 1:10000) from Cell Signaling Leiden, The Netherlands, anti-EGFR (1005, sc-03-G, 1:1000) from Santa Cruz Biotechnology Inc., and anti-CXCR7 (GTX100027, 1:1000) from GeneTex, Irvine, CA, USA—followed by treatment with the secondary antibodies goat anti-rabbit HRP (#P0448, 1:2000) or rabbit anti-mouse HRP (#P0260, 1:2000), depending on the primary antibody, from DakoCytomation, Glostrup, Denmark, for 1 h at RT. The bands were visualized using the Western Lightning Plus-ECL kit (PerkinElmer, Waltham, MA, USA) and quantified by GeneSnap image analysis software (SynGene, Frederick, MD, USA). 

### 2.5. DNA Isolation, Polymerase Chain Reaction (PCR), and Sequencing

Cells were harvested by trypsin and washed with PBS. DNA was isolated using the DNeasy Blood & Tissue Kit (Qiagen, Hilden, Germany) according to the manufacturer’s protocol. Exons 2 and 3 of the *EGFR* were amplified by PCR using standard procedures. PCR was performed using 150 ng of genomic DNA in a final volume of 25 µL containing 1× PCR buffer, 0.25 µL of Pfu DNA polymerase (Thermo Fisher Scientific, Waltham, MA, USA) and 100 nM primers (Table S2). The PCR program comprised one cycle of 98 °C for 2 min, 30 cycles of 98 °C for 10 s, of 59 °C for 15 s, and of 72 °C for 30 s, and one cycle of 72 °C for 10 min. PCR products were analyzed on 1.5% agarose gel. PCR products were purified using the Wizard SV Gel and PCR Clean-Up Kit (Promega, Madison, WI, USA) according to the company’s protocol and sequenced at Macrogen Europe (Amsterdam, The Netherlands).

### 2.6. Colony Formation Assay

Total numbers of 500 or 1000 cells were seeded in a single well of a 6-well plate and treated with 100 nM cetuximab or 5 μM gefitinib for 12 days at 37 °C in a humidified CO_2_ incubator. The medium was then removed and cells were washed with PBS, followed by cell fixation using 4% formaldehyde. Cells were stained with 1% crystal violet and colonies were counted. Experiments were performed in triplicate and repeated at least three times.

To test the effects of EGF and CXCR7 inhibitor on cell proliferation, 10,000 cells were seeded in a 12-well plate and grown for 6 days at 37 °C in a humidified CO_2_ incubator. The medium was removed, and cells were washed with PBS, followed by cell fixation using 4% formaldehyde. Cells were stained with 1% crystal violet. For quantification of the staining, 1 mL of 10% acetic acid was used for each well to extract the dye, and the absorbance was measured at wavelength of 590 nm.

### 2.7. Wound Healing Assay

The A549 *EGFR^wt/wt^* and *EGFR*^−/−^ cells were seeded in 6-well plates at a density of 7 × 10^5^ cells per well and starved overnight in DMEM containing 1% FBS. A wound was gently made by scraping the cells with a sterile 200 μL pipette tip. The detached cells were removed using PBS. Pictures were taken at 0 h, 12 h, and 24 h using a CK2 inverted microscope (Olympus, Tokyo, Japan). Experiments were performed in triplicate and repeated at least three times.

### 2.8. Cell Migration Assay

A total number of 1 × 10^4^ cells in DMEM containing 1% FBS was added to the top Transwell insert. The 24-well plate wells were filled with a 750 μL culture medium. The Transwell insert was gently added to the 24-well plate and incubated at 37 °C with 5% CO_2_ for 20–24 h. Medium and remaining cells were carefully removed from the top of the membrane. The insert membrane was stained with 0.5% crystal violet. Invaded cells were photographed randomly and counted. Experiments were performed in triplicate and repeated at least three times.

### 2.9. MTS Assay

A total of 3 × 10^3^ cells were seeded per well in 96-well plates and cultured for 24 h. Cells were then treated with appropriate drugs for 72 h. Next, cells were incubated at 37 °C with a medium containing MTS for 90 min following the protocol of CellTiter 96 AQueous One Solution Reagent (Promega, Madison, WI, USA). The absorbance was determined at a wavelength of 490 nm using a Synergy H1 plate reader (BioTek, Winooski, VT, USA). Experiments were performed in triplicate and repeated at least three times.

### 2.10. Flow Cytometric Analysis

Cells were harvested, washed twice with standard FACS buffer (PBS/1%FBS), and incubated with a primary antibody (cetuximab) or a human IgG isotype as a negative control (Invitrogen, Carlsbad, CA, USA) for 1 h on ice. Cells were then washed with FACS buffer, followed by 1 h incubation with a goat anti-human IgG (H + L) Cross-Adsorbed secondary antibody, Alexa Fluor 488. EGFR membrane expression was determined using a FACSCalibur flow cytometer (BD, Franklin Lakes, NJ, USA).

### 2.11. RNA Isolation and Quantitative Reverse Transcriptase PCR (qRT-PCR)

Cells were harvested by trypsin and washed with PBS. RNA was isolated using the Maxwell LEV simply RNA Cells/Tissue Kit (Promega, Madison, WI, USA). RNA concentrations were measured using NanoDrop (Thermo Fisher Scientific, Waltham, USA). cDNA was synthesized from 200 ng of total RNA using the Reverse Transcription kit (Promega, Madison, WI, USA) according to the manufacturer’s instruction.

qRT-PCR was performed using 20 ng of cDNA as input and SensiMix SYBRkit (Bioline, Taunton, MA, USA) in an ABI Prism 7900HT Sequence Detection System (Thermo Fisher Scientific, Waltham, MA, USA). Primer sets are listed in Table S2. Data analysis was performed using SDS v.2.3 software (Applied Biosystems, Foster City, CA, USA). Glyceraldehyde-3-phosphate dehydrogenase (GAPDH) mRNA levels were measured and used as reference for data normalization.

### 2.12. CXCR7 Knockdown Using siRNAs

Approximately 3 × 10^5^ cells per well were cultured for 24 h in a 6-well plate. Cells were then transfected with a mixture of CXCR7-specific validated siRNAs (Thermo Fisher Scientific, Waltham, USA) and negative control siRNAs (Thermo Fisher Scientific, Waltham, WI, USA) using Lipofectamine 3000 (Invitrogen, Carlsbad, USA) according to the manufacturer’s protocol. Total RNA was isolated 72 h after transfection. Experiments were repeated three times.

### 2.13. EGFR Rescue in A549 EGFR^−/−^ Cells

A total of 3 × 10^5^ A549 *EGFR*^−/−^ cells per well were cultured for 24 h in a 6-well plate. Cells were transfected with pCDNA6A-*EGFR* wild-type plasmids using Lipofectamine 3000 (Invitrogen, Carlsbad, USA). Culture medium was changed one day after transfection, followed by blasticidin selection with an appropriate concentration for the next three days. EGFR expression level was determined by Western blot. Experiments were repeated three times. pCDNA6A-EGFR wild-type plasmid was a gift from Mien-Chie Hung (Addgene plasmid #42665).

### 2.14. Patient Tumor Samples and Immunohistochemistry (IHC)

A total of 47 patients with lung cancer were included in the study. A tissue microarray (TMA) containing 43 formalin fixed paraffin embedded (FFPE) primary lung tumor samples from the Department of Pathology, University Medical Center Groningen (UMCG), and 4 additional FFPE primary lung tumor samples from The First Hospital of Lanzhou University were used to detect CXCR7 protein expression using IHC. The study was performed in accordance with the Declaration of Helsinki and Good Clinical Practice guidelines.

Briefly, 4 μm FFPE sections were deparaffinized using xylene for 10 min. Next, slides were incubated with Tris/HCl (pH = 9) in a microwave. After blocking endogenous peroxidase activity with hydrogen peroxide, slides were incubated with the anti-CXCR7 primary rabbit polyclonal antibody (GTX100027, GeneTex, Irvine, USA) for 1 h at RT. Slides were then incubated with peroxidase-labeled goat anti-rabbit secondary and rabbit anti-goat tertiary antibodies (Dako, Denmark) for 30 min at RT. Visualization was performed using ImmPACT NovaRED Peroxidase (HRP) Substrate (Vector Laboratories, Burlingame, CA, USA) followed by hematoxylin staining. Scoring of the slides was performed by an experienced pulmonary pathologist (WT). 

### 2.15. Statistical Analysis

The data are presented as mean ± SD, except where otherwise indicated. Data are derived from three or more independent experiments (unless otherwise indicated), and statistical analysis was performed using GraphPad software v.5.0 (GraphPad Software, La Jolla, CA, USA). Results were analyzed by a two-tailed unpaired student’s *t*-test unless otherwise noted. A chi-square test was used to determine significance in IHC results. *p*-values less than 0.05 were considered significant. The synergic analysis was performed using CompuSyn Program (ComboSyn, Paramus, NJ, USA).

## 3. Results

### 3.1. EGFR Knockout in A549 Cell Line

**Figure 1:**
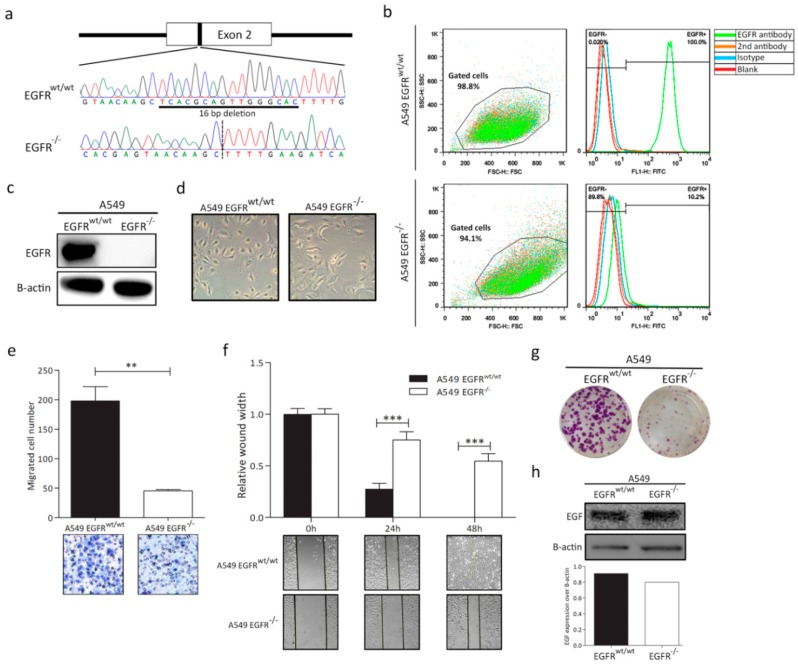
CRISPR/Cas9-mediated epidermal growth factor receptor (*EGFR*) knockout in non-small cell lung cancer (NSCLC) A549 cells and characterization of A549 *EGFR^wt/wt^* and *EGFR*^−/−^ cells. (**a**) Schematic diagram of guide RNAs (gRNAs) targeting exon 2 of the *EGFR* gene in A549 cells and validation by Sanger sequencing. (**b**) FACS analysis shows EGFR expression in *EGFR^wt/wt^* and *EGFR*^−/−^ cells. (**c**) Western blot demonstrating EGFR expression in *EGFR^wt/wt^* and absence of EGFR protein in *EGFR*^−/−^ cells. (**d**) Morphologic changes of *EGFR^wt/wt^* and *EGFR*^−/−^ cells. Magnification: 10x(**e**) Cell migration analysis using a Transwell assay. (**f**) Wound healing assay to evaluate wound closure and cell migration ability at different time points. (**g**) Colony formation of *EGFR^wt/wt^* and *EGFR*^−/−^ cells. (**h**) Western blot showing EGF expression in A549 *EGFR^wt/wt^* and *EGFR*^−/−^ cells. Data in bar graphs are represented as mean ± SD (*n* ≥ 3); two-tailed unpaired student’s *t*-test: * *p*-values < 0.05; ** *p*-values < 0.01; *** *p*-values < 0.001; ns: not significant.

A549 is an adenocarcinoma cell line that contains a wild-type *EGFR* and mutant *KRAS*. To generate an A549 *EGFR* knockout cell lines, a CRISPR/Cas9 approach was applied using three gRNAs targeting exons 2 and 3 of *EGFR* (Table S1). Sanger sequencing revealed a 16 bp deletion in exon 2 of *EGFR*, which resulted in a premature stop codon, L79X (Figure 1a). Another independent clone with 1 bp insertion in exon 3 was shown in Figure S1. FACS and Western blot showed expression of EGFR in the wild-type A549 cells, while it was totally absent in the *EGFR* knockout cell line (Figure 1b,c and Figure S2). 

### 3.2. EGFR Plays a Significant Role in Cell Progression in KRAS-Dependent Lung Cancer Cells

To characterize the phenotypic effect of *EGFR* loss in KRAS-dependent NSCLC cells, we examined the proliferation and migration ability of the A549 *EGFR^wt/wt^* and *EGFR*^−/−^ cells. We observed the morphological changes in *EGFR*^−/−^ cells. Compared to the normal spindle shaped A549 cells, A549 *EGFR*^−/−^ cells showed a more flat and irregular polygonal shape (Figure 1d). A549 *EGFR*^−/−^ cells showed significant attenuated proliferation and migration properties (Figure 1e–g and Figure S3). To exclude the interference of endogenous EGF, we examined the expression of EGF by Western blot. We did not observe any obvious change in EGF levels in A549 *EGFR^wt/wt^* and *EGFR*^−/−^ cells (Figure 1h).

### 3.3. EGFR Knockout Does Not Significantly Affect Drug Sensitivity

**Figure 2:**
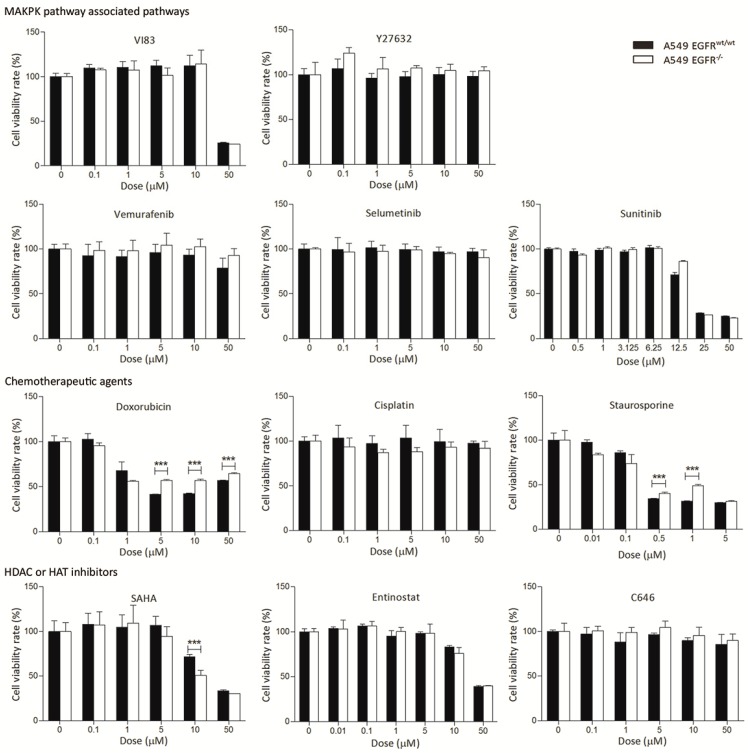
Treatment of the A549 *EGFR^wt/wt^* and *EGFR*^−/−^ cells with different drugs. Cells were treated with drugs at indicated concentrations for 72 h and cell viability was determined with an MTS assay. For staurosporine, cell viability was determined after 24 h due to its toxicity. Data in bar graphs are represented as mean ± SD (*n* ≥ 3); two-tailed unpaired student’s *t*-test: * *p*-values < 0.05; ** *p*-values < 0.01; *** *p*-values < 0.001; ns: not significant.

To determine drug response changes in A549 *EGFR^wt/wt^* and *EGFR*^−/−^ cells, we tested a series of targeted and chemotherapy drugs, including anti VEGFR, BRAF, PDGFR, MEK, and HDAC/HAT, cisplatin, doxorubicin, and staurosporine. At low to intermediate doses of the drugs, we did not observe substantial differences between *EGFR^wt/wt^* and *EGFR*^−/−^ cells (Figure 2). At higher levels, A549 *EGFR*^−/−^ cells showed slightly more resistance to doxorubicin and cisplatin than *EGFR^wt/wt^* cells did. We also observed more resistance to staurosporine, a well-known apoptosis inducer, in A549 *EGFR*^−/−^ cells (Figure 2). However, the *EGFR*^−/−^ cells were more sensitive to SAHA as compared to the *EGFR^wt/wt^* cells at a dose of 10 µM. 

### 3.4. Effect of EGFR Loss on Downstream Pathways

**Figure 3:**
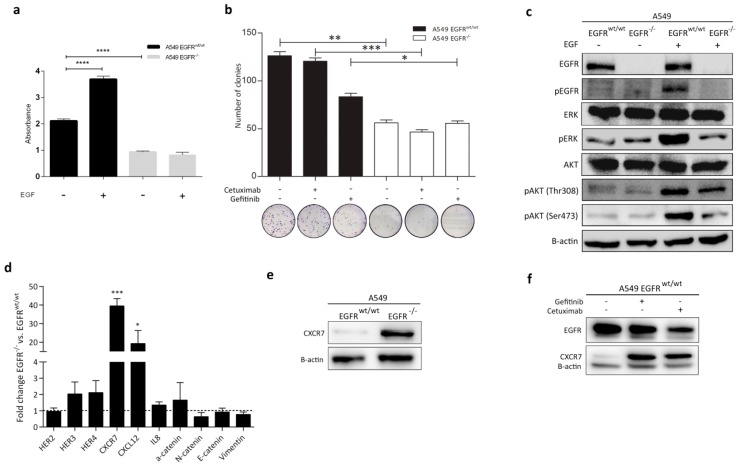
Effect of different treatments on cell proliferation and expression of EGFR downstream signaling proteins in A549 *EGFR^wt/wt^* and *EGFR*^−/−^ cells. (**a**) Proliferation analysis of *EGFR^wt/wt^* and *EGFR*^−/−^ cells in the presence and absence of EGF. Cells were cultured in serum-starved medium (2% serum). (**b**) Colony formation ability of *EGFR^wt/wt^* and *EGFR*^−/−^ cells before and after treatment. (**c**) Western blot analysis of EGFR downstream proteins before and after stimulation with EGF for 15 min. (**d**) Relative mRNA expression of the selected genes. (**e**) Western blot analysis of CXCR7 in *EGFR^wt/wt^* and *EGFR*^−/−^ cells. (**f**) CXCR7 protein expression in *EGFR^wt/wt^* cells treated with 0.5 µM gefitinib and 100 nM cetuximab for 72 h. Data in bar graphs are represented as mean ± SD (*n* ≥ 3); two-tailed unpaired student’s *t*-test: * *p*-values < 0.05; ** *p*-values < 0.01; *** *p*-values < 0.001.

To test the effect of EGF on cell growth, we performed a clonogenic proliferation assay in the presence and absence of EGF in A549 *EGFR^wt/wt^* and *EGFR*^−/−^ cells. As expected, EGF can stimulate *EGFR^wt/wt^* cell proliferation but did not affect it in *EGFR*^−/−^ cells (Figure 3a). Thus, *EGFR* loss resulted in decreased cell proliferation with or without EGF (Figure 3a). *EGFR* loss also led to a stronger inhibition of cell proliferation as compared to gefitinib and cetuximab treatment (Figure 3b). As EGFR plays crucial roles in the PI3K-Akt and MAPK/ERK pathways, we determined changes in these two key pathways after *EGFR* loss. We investigated expression of EGFR, pEGFR, Akt, pAkt (Thr308 and Ser473), and MAPK (pERK) by Western blot. After stimulation with EGF, the induction of pEGFR, pERK, and pAkt (Thr308 and Ser473) was substantially lower in A549 *EGFR*^−/−^ cells in comparison with *EGFR^wt/wt^* cells (Figure 3c). In contrast, pERK levels were slightly higher in A549 *EGFR*^−/−^ cells than the wild-type cells in the absence of EGF (Figure 3c). 

### 3.5. CXCR7 Is Significantly Upregulated in A549 EGFR^−/−^ Cells and EGFR^wt/wt^ Cells Treated with EGFR Inhibitors

We performed qRT-PCR on a panel of genes associated with the EGFR/MAPK pathway and epithelial-mesenchymal transition (EMT) (Table S2). Surprisingly, CXCR7 showed a marked upregulation at the RNA and protein levels in A549 *EGFR*^−/−^ cells (Figure 3d,e and Figure S4). In addition, we observed overexpression of CXCL12 (19-fold), a main ligand for CXCR7, in *EGFR*^−/−^ cells (Figure 3d). Treatment of the *EGFR^wt/wt^* cells with gefitinib or cetuximab for 72 h resulted in an increased CXCR7 expression level (Figure 3f). This shows that inhibition or loss of EGFR induces CXCR7 expression in A549 cells and may subsequently contribute to tumor survival.

In contrast to CXCR7, moderate changes were observed in the expression of *HER3* (twofold higher) and *HER4* (twofold higher) in A549 *EGFR*^−/−^ cells as compared to *EGFR^wt/wt^* cells (Figure 3d). Differences in *HER2* mRNA levels were negligible. We did not observe any significant changes in the expression of *EMT*-related markers. Only the levels of α-catenin mRNA were slightly increased by 1.6-fold in A549 *EGFR*^−/−^ cells (Figure 3d).

### 3.6. Dual Inhibition of CXCR7 and EGFR Downregulates MAPK (ERK1/2) and Suppresses Proliferation

**Figure 4:**
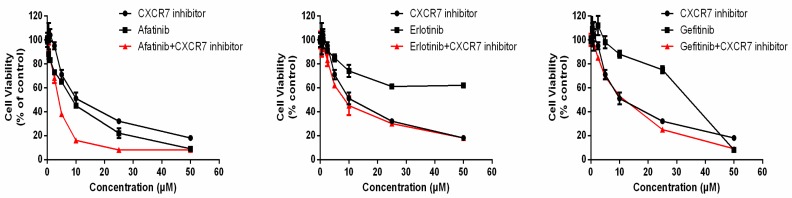
Dual inhibition of CXCR7 and EGFR. Combination of afatinib and CXCR7 inhibitor induces a synergistic inhibitory effect in A549 cell line. Cells were treated with drugs at indicated concentrations for 72 h, and cell viability was determined with an MTS assay.

We investigated the synergistic inhibitory effect of gefitinib, erlotinib, and afatinib with a CXCR7 inhibitor. We observed a significant synergistic inhibitory effect of CXCR7 inhibitor combined with afatinib (Figure 4 and Table 1). However, a combination of CXCR7 inhibitor with erlotinib or gefitinib did not show an obvious inhibitory effect (Figure 4).

**Figure 5:**
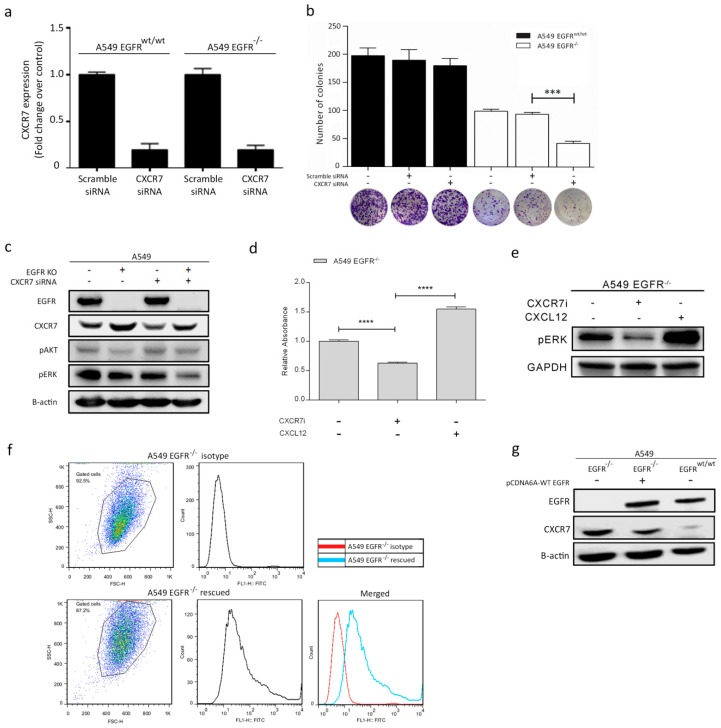
The interaction of EGFR and CXCR7 and their effect on downstream pathways and cell proliferation in A549 *EGFR^wt/wt^* and *EGFR*^−/−^ cells. (**a**) CXCR7 mRNA levels after transient transfection with either scramble siRNA or CXCR7 siRNA. (**b**) Colony formation assay to determine the effect of CXCR7 knockdown on cell proliferation. (**c**) Western blot analysis of CXCR7, pAKT, and pERK protein expression levels after CXCR7 knockdown. (**d**) Colony formation assay to determine the effects of the CXCR7 inhibitor and CXCL12 on cell proliferation. (**e**) Western blot analysis of pERK after treatment of *EGFR*^−/−^ cells with CXCR7 inhibitor (2 μM) for 24 h and CXCL12 (100 ng/mL) for 3 min. (**f**) FACS analysis shows EGFR expression in A549 *EGFR*^−/−^ following transfection with *EGFR* pCDNA. (**g**) Western blot analysis of EGFR and CXCR7 in A549 *EGFR*^−/−^ cells transfected with EGFR pCDNA, *EGFR*^−/−^, and *EGFR^wt/wt^* cells. Data in bar graphs are represented as mean ± SD (*n* ≥ 3); two-tailed unpaired student’s *t*-test: * *p*-values < 0.05; ** *p*-values < 0.01; *** *p*-values < 0.001; ns: not significant.

Next, we knocked down CXCR7 by siRNAs in A549 *EGFR^wt/wt^* and *EGFR*^−/−^ cells to explore whether cell proliferation can be further suppressed (Figure 5a,b). We showed that CXCR7 knockdown alone did not change A549 *EGFR^wt/wt^* cell growth. However, CXCR7 knockdown significantly decreased proliferation of A549 *EGFR*^−/−^ cells (Figure 5b).

To gain insight into the CXCR7-dependent mechanisms that lead to tumor cell proliferation, we checked MAPK (ERK1/2) expression. *CXCR7* knockdown decreased pERK levels in A549 *EGFR*^−/−^ cells as compared to *EGFR^wt/wt^* cells (Figure 5c). Moreover, CXCR7 inhibition can further restrain the proliferation of *EGFR*^−/−^ cells (*p* < 0.001) (Figure 5d) and decrease pERK levels (Figure 5e). Conversely, stimulation of CXCR7 with its ligand CXCL12 promoted proliferation of A549 *EGFR*^−/−^ cells (Figure 5d) and increase pERK levels (Figure 5e). Taken together, our results suggest that CXCR7 induces proliferation by enhancing pERK levels in A549 *EGFR*^−/−^ cells.

### 3.7. Rescue of EGFR Leads to CXCR7 Downregulation in A549 EGFR^−/−^ Cells

To further confirm the interaction between EGFR and CXCR7, we rescued EGFR in A549 *EGFR*^−/−^ cells. Overexpression of wild-type *EGFR* in A549 *EGFR*^−/−^ cells restored EGFR membrane expression (Figure 5f). In the control groups, EGFR signals were not detected. Interestingly, CXCR7 expression dramatically decreased after the reintroduction of EGFR to levels slightly above those in *EGFR^wt/wt^* cells (Figure 5g). These data suggest a tight functional regulatory link between EGFR and CXCR7.

### 3.8. CXCR7 Expression, Altered by the Ablation of EGFR, Is Associated with the Genotype of NSCLC

**Figure 6:**
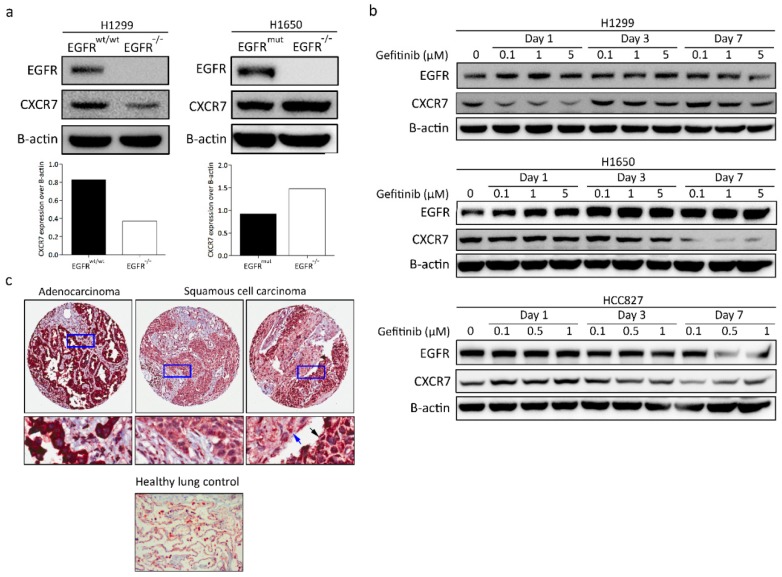
CXCR7 expression in different NSCLC cell lines with or without gefitinib treatment and CXCR7 expression in NSCLC patients. (**a**) Western blot analysis of H1299, H1650 *EGFR^wt/wt^*, and *EGFR*^−/−^ cells. (**b**) Western blot analysis of H1299, H1650, and HCC827 cells treated with different doses of gefitinib over time. (**c**) Immunohistochemical staining of CXCR7 in NSCLC primary tumor samples (*n* = 47). The blue and black arrows show the expression of CXCR7 in mature and less differentiated squamous cell lung carcinoma (SQCC), respectively.

We next determined whether CXCR7 shows similar effects in lung cancer cells with different genetic backgrounds. Knockout of *EGFR* in H1299 cells, with wild-type *EGFR,* led to a dramatic decrease in CXCR7 levels (Figure 6a). Knockout of *EGFR* in H1650, with exon 19 deletions in *EGFR*, revealed higher expression of CXCR7.

Next, we treated H1299, H1650, and HCC827 cells with gefitinib to investigate changes in the expression levels of CXCR7. H1299 is resistant to gefitinib (IC50, 40 µmol/L) [32]. In contrast to the H1299 *EGFR*^−/−^ cells, H1299 parental cells showed a decreased CXCR7 expression level after one-day treatment with gefitinib and an increased expression with a low dose of gefitinib three and seven days after the start of treatment (Figure 6b). 

Interestingly, both CXCR7 and EGFR showed a highly dose- and time-dependent expression pattern in H1650 cells after treatment with gefitinib (Figure 6b). Moreover, EGFR and CXCR7 expression showed a clear negative correlation upon gefitinib treatment. Particularly, CXCR7 expression was decreased, whereas EGFR expression increased after treatment with different doses of gefitinib over time in H1650 (Figure 6b). Both EGFR and CXCR7 expression increased after one day of treatment with gefitinib (Figure 6b). However, in *EGFR*-mutant NSCLC cell lines (HCC827 and H1650), EGFR and CXCR7 expression substantially decreased after longer treatment (three and seven days) with gefitinib in a dose-dependent manner (Figure 6b). Thus, in *EGFR*-mutant NSCLC cell lines (HCC827 and H1650), CXCR7 expression levels decreased with gefitinib treatment. Conversely, in *EGFR* wild-type NSCLC cell lines (H1299 and A549), CXCR7 expression levels increased after gefitinib treatment (Figure 3f and Figure 6b). Together, these data indicate that CXCR7 and EGFR have a close interaction that is dependent on the *EGFR* genotype. 

### 3.9. CXCR7 Is Highly Expressed in Primary Lung Adenocarcinoma

To investigate the clinical relevance of CXCR7 expression in NSCLC patients, we evaluated CXCR7 expression in 47 primary NSCLC patients by IHC. Immunostaining revealed strong cytoplasmic expression of CXCR7 in 74.5% (35/47), weak expression in 17% (8/47), and heterogeneous expression (weak and strong) in 8.5% (4/47) of the tumor samples (Figure 6c and Table 2). CXCR7 expression was weak in the healthy lung tissue (Figure 6c). 

CXCR7 expression was strong in the vast majority of the ADCs (92%). In contrast to ADC cases, only 40.5% of SQCC tumors showed strong CXCR7 expression. Approximately 33.3% had a weak expression, and 22.2% of the SQCC tumors showed an intra-heterogeneous CXCR7 expression pattern. In this heterogeneous group, the expression of CXCR7 was weaker in more differentiated tumor cells as compared to the less differentiated ones (Figure 6c and Table 2). Other subtypes (NSCLC NOS and pleomorphic carcinoma; *n* = 4) showed strong expression. These results suggest that CXCR7 may play a role in the development of different histological subtypes of NSCLC tumors.

## 4. Discussion

*KRAS* and *EGFR* are commonly mutated in NSCLC. However, whether targeting EGFR is an option for the treatment of KRAS-dependent NSCLC is not clear. In this study, we showed that EGFR plays a critical role in the proliferation of KRAS-dependent cells. Furthermore, we report a novel genotype-dependent crosstalk between EGFR and CXCR7 in NSCLC cells. We showed that EGFR disruption or blockage can lead to upregulation of CXCR7 and activation of the MAPK signaling pathway, resulting in outgrowth or survival advantage of the KRAS-dependent cells. We also demonstrated that dual inhibition of EGFR and CXCR7 suppressed tumor cell growth and reduced MAPK activity by downregulation of pERK levels. Dual inhibition of EGFR and CXCR7 by inhibitors leads to a synergistic effect, which may provide a novel therapeutic approach for NSCLC patients. We also showed that CXCR7 is overexpressed in ADC patients as compared to the SQCC cases. 

*KRAS*-mutant tumor cells appear to rely on KRAS for cell proliferation [33]. In this study, we showed that EGFR also plays an essential role in the survival of NSCLC cells harboring *KRAS* mutations. We also observed morphological changes in *EGFR*^−/−^ lung tumor cells as well as substantial reduction in migration ability of these cells. It is not surprising that previous studies have shown that gefitinib is not efficient for the treatment of NSCLC patients with wild-type *EGFR*, because gefitinib has a higher affinity to the mutated version of EGFR, such as L858R point mutation, as compared to wild-type EGFR [34]. Anti-EGFR therapy with EGFR blocking antibody cetuximab shows resistance due to dysregulation of EGFR and activation of other HER family receptors [35]. Thus, these studies do not show that wild-type EGFR is not necessary for tumor proliferation. Based on our results, wild-type EGFR is important for proliferation and migration of *KRAS*-mutant tumor cells.

We used 11 inhibitors, including MAPK associated inhibitors, chemotherapeutic agents, HDAC and HAT inhibitors, and an apoptosis inducer agent to evaluate the drug response in A549 *EGFR^wt/wt^* and *EGFR*^−/−^ cells. However, we did not observe significant differences between the two genotypes. These results indicate the ineffectiveness of clinically available drugs for the treatment of *KRAS*-mutant NSCLC cells. In contrast, simultaneous inhibition (inhibitors or knockdown) of EGFR and CXCR7 significantly attenuated cancer cell growth. Based on our results, we propose a potential novel strategy for the treatment of KRAS-dependent NSCLC by dual inhibition of EGFR and CXCR7. Further in vivo studies are needed to identify appropriate delivery methods that can efficiently and specifically target EGFR and CXCR7 in tumor cells [36,37].

CXCR7 contributes to angiogenesis and cell growth in lung tumors [21,23,38,39]. In addition, overexpression of CXCR7 was associated with poorer prognosis in lung cancer patients [33]. The latter effect was probably induced by the activation of the MAPK pathway [28,40]. We showed that EGFR blockage or knockout leads to reduced growth and a significant increase in CXCR7 levels in A549 cells. CXCR7 knockdown decreased pERK levels and further suppressed proliferation of A549 *EGFR*^−/−^ cells. Dual inhibition of EGFR and CXCR7 by inhibitors can achieve a synergistic inhibitory effect. Thus, our observations suggest that CXCR7 overexpression is one of the survival mechanisms in KRAS-dependent tumor cells with EGFR loss or cells treated with EGFR-TKIs. Interestingly, we only observed the inhibitory effect of concomitant combination of CXCR7 inhibitor and afatinib, but not gefitinib or erlotinib. One possible explanation is that afatinib is an irreversible HER family inhibitor. Gefitinib and elortinib only have a strong inhibitory effect on mutant *EGFR*, but afatinib can effectively inhibit both wild-type and mutant *EGFR* [41,42]. It has previously been shown that afatinib can suppress tumor growth in *KRAS*-mutant NSCLCs, but not erlotinib or gefitinib. Interestingly, our in vitro results are in concordance with a previously published in vivo study [43]. These observations demonstrate that dual inhibition of CXCR7 and EGFR have a synergistic therapeutic effect in *KRAS*-mutant NSCLC cells. 

CXCL12 is involved in cell proliferation and brain metastasis in lung cancer patients [39]. CXCL12 is overexpressed in NSCLC cell lines and primary lung cancer [44] and may promote tumor cell migration and growth through the ERK pathway [45]. We observed an increased expression of CXCL12, a shared ligand of CXCR7 and CXCR4 with a higher affinity towards CXCR7, in A549 *EGFR*^−/−^ cells. CXCR7 knockdown is highly efficient in the suppression of cell proliferation of A549 *EGFR*^−/−^ cells, as shown in our study. However, other ligands of CXCR7 such as CXCL11, MIF, and Dickkopf-3 may also influence CXCR7-mediated cell proliferation, which requires further investigation. We did not observe significant changes in the expression levels of other HER family receptors including HER2, HER3, and HER4 in A549 *EGFR*^−/−^ cells. Thus, our data suggest that HER family member receptors are not significantly affected by EGFR loss. 

Our results show that MAPK/pERK is continuously activated in both *EGFR*^−/−^ and *EGFR^wt/wt^* cells. This is indicative of an EGFR-independent survival mechanism, which may be partly explained by the presence of *KRAS* activating mutations in these cells. *KRAS* mutations can lead to constant activation of RAS/RAF/MEK/ERK [46]. Furthermore, as EGFR is upstream of the RAS pathway, loss of EGFR may not affect activation of RAS/RAF/MEK/ERK introduced by mutant *KRAS*. Notably, one study showed that KRAS pathways coordinate the transduction of CXCL12/CXCR4/CXCR7 and influence growth of pancreatic cancer [47]. It will be of great interest to explore whether CXCR7 expression, altered by the ablation of EGFR, is associated with KRAS in NSCLC. We showed that EGF can still stimulate pAkt in *EGFR*^−/−^ cells. This observation indicates that EGF-induced phosphorylation of Akt may be independent of EGFR, which to our knowledge has not been reported previously. One hypothesis is that EGF stimulates pAkt via CXCR7, because previous studies have shown that CXCR7 associated pathways can induce Akt phosphorylation [48,49], and we observed CXCR7 overexpression in A549 *EGFR*^−/−^ cells. In addition, one recent study reported that Akt activation is related to EGFR drug resistance in lung cancer [50]. Given the importance of Akt for tumor survival and growth [51], future studies are needed to explore the crosstalk between EGF-EGFR and CXCL12-CXCR7 in the MAPK pathway.

**Figure 7:**
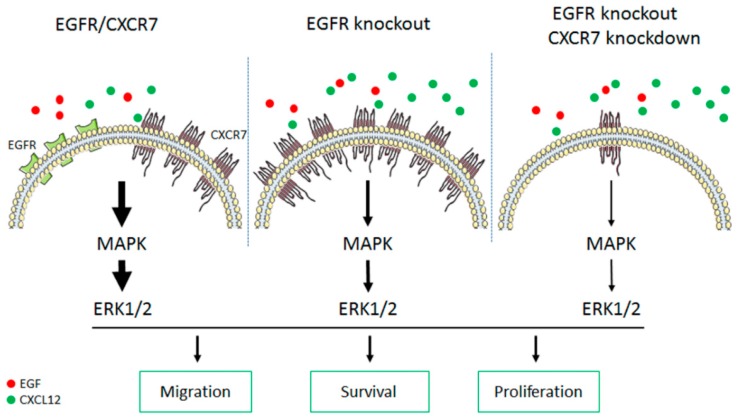
The proposed model of CXCR7-EGFR crosstalk and its effect on the MAPK signaling pathway. EGFR-ERK and CXCR7-ERK signaling pathways promote tumor cell development (left). Ablation of EGFR leads to overexpression of CXCR7/CXCL12 and subsequently stimulates the ERK pathway, resulting in tumor cell survival (middle). Dual inhibition of EGFR and CXCR7 further suppresses ERK signaling and synergistically inhibits tumor growth (right). Thickness of the arrow shows strength of the signaling. The number of solid dots (in red or green) shows the amount of the endogenous epidermal growth factor (EGF) or CXCL12.

We found a close inverse relationship between EGFR and CXCR7 expression (Figure 7). When EGFR is expressed in the A549 *EGFR*^−/−^ cells, a decreased expression of CXCR7 is observed. Mechanisms leading to CXCR7 upregulation in *EGFR*^−/−^ cells are still not clear. Previous studies showed that the CXCR7 promoter contains five NF-kB binding sites and activated NF-kB can upregulate CXCR7 [52]. Thus, one potential mechanism can be that CXCR7 is transcriptionally activated by NF-kB. Several studies have shown that NF-kB activation drives survival of tumor cell treated with EGFR-TKIs [53,54,55,56]. Another possibility is that CXCR7 overexpression may be related to the mutational status of *KRAS* [47]. 

We showed that upon treatment with gefitinib, CXCR7 expression dramatically decreased in *EGFR*-mutant NSCLC cell lines, but, conversely, it is increased in *EGFR* wild-type NSCLC cell lines. Blocking mutant EGFR with TKIs can substantially inhibit MAPK/ERK and suppress tumor cell proliferation [57,58]. This indicates that EGFR-TKIs may reduce pERK and inhibit tumor cell proliferation partially through downregulation of CXCR7. Moreover, insensitivity or acquired resistance of *EGFR* wild-type NSCLC cells for TKIs might be due to increased CXCR7 levels. Our results show that CXCR7 expression is highly related to the genetic background of the lung cancer cells, at least in the three cell lines we tested, suggesting the importance of personalized therapy in lung cancer treatment. Importantly, we observed higher expression of CXCR7 in lung ADCs (92% strongly expressed CXCR7) as compared to lung SQCC cases (40.5% strongly expressed CXCR7). One study, which performed a meta-analysis in nearly 3000 patients, showed that EGFR was less expressed in ADC (39%) than in SQCC (58%) [59]. Given this study and our results, there may be a correlation between EGFR and CXCR7 in patients with NSCLC. Moreover, higher expression of CXCR7 in the less differentiated SQCC tumors may be due to its critical role during tumor initiation and the differentiation process of this tumor subtype. Higher expression of CXCR7 in lung cancer tissues as compared to the healthy lung tissue suggests CXCR7 may be a target for lung cancer treatment. 

## 5. Conclusions

Our data show that wild-type *EGFR* plays a significant role in *KRAS*-mutant NSCLC cancer cells and revealed CXCR7 upregulation as a potential survival mechanism in *KRAS*-mutant cells upon EGFR loss (Figure 7). Our findings suggest that dual inhibition of EGFR and CXCR7 may be a promising therapeutic strategy for at least a subset of patients with NSCLC. 

## Figures and Tables

**Table 1:** Effect of concomitant combination of EGFR-TKI (TKI: tyrosine kinase inhibitor) and CXCR7 inhibitor on A549 cells based on the Chou and Talalay method.

Combination Treatment	TKIs (µM)	CXCR7i (µM)	FA	CI	Effect
Afatinib + CXCR7i	10	10	0.84	0.47	Synergistic
Erlotinib + CXCR7i	10	10	0.55	0.74	Moderately Synergistic
Gefitinib + CXCR7i	10	10	0.47	>1.1	Antagonistic

FA represents the fraction of growth effect of drug-treated cells compared with control cells and CI represents the combination index. CI = 1: additivity; CI > 1: antagonism; CI < 1: synergism.

**Table 2:** CXCR7 expression in lung cancer patients.

Subtype	Strong (%)	Weak (%)	Strong-Weak (%)	Total	*p*-Value
Histology					0.005 *
ADC	23 (92)	2 (8)	0	25	
SQCC	8 (44.5)	6 (33.3)	4 (22.2)	18	
Other	4 (100)	0	0	4	

* Chi-square test.

## Supplementary Materials

The following are available online at https://www.mdpi.com/2072-6694/11/4/455/s1, Figure S1: Schematic diagram of gRNAs targeting exon3 of the EGFR gene in A549 cells and validation by Sanger sequencing for clone 1, Figure S2: Western blot demonstrating EGFR expression in EGFR*^wt/wt^* and absence of EGFR protein in EGFR^−/−^ cells (Clone 1), Figure S3: Wound healing assay to evaluate wound closure and cell migration ability at different time points (Clone 1), Figure S4: qPCR and Western blot analysis of CXCR7 in A549 EGFR*^wt/wt^* and EGFR^−/−^ cells (Clone 1), Table S1: List of gRNA sequences for CRISPR/Cas9, Table S2: List of the primer sets used for qRT-PCR.
