# Supplementary material for: CX Chemokine Receptor 7 Contributes to Survival of KRAS-Mutant Non-Small Cell Lung Cancer upon Loss of Epidermal Growth Factor Receptor
Source: Cancers (Basel). 2019 Mar 30;11(4):455. doi: 10.3390/cancers11040455 (PMC6520904; doi:10.3390/cancers11040455)
Supplement: Supplementary file 1 [file cancers-11-00455-s001.pdf]

cancers

Supplementary Materials: CX Chemokine Receptor 7 Contributes to Survival of *KRAS*-Mutant Non-Small Cell Lung Cancer Cells upon Loss of Epidermal Growth Factor Receptor

Bin Liu, Shanshan Song, Rita Setroikromo, Siwei Chen, Wenteng Hu, Deng Chen, Anthonie J. van der Wekken, Barbro N. Melgert, Wim Timens, Anke van den Berg, Ali Saber and Hidde J. Haisma

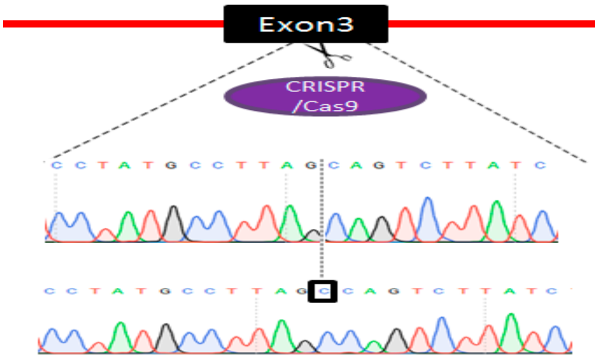

Figure S1. Schematic diagram of gRNAs targeting exon3 of the EGFR gene in A549 cells and validation by Sanger sequencing for clone 1.

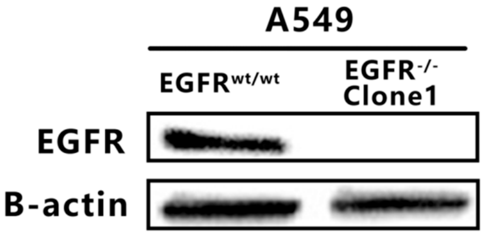

Figure S2. Western blot demonstrating EGFR expression in $EGFR^{wt/wt}$ and absence of EGFR protein in $EGFR^{-/-}$ cells (Clone 1).

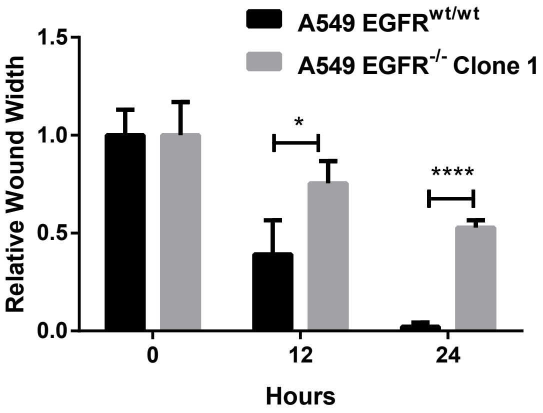

Figure S3. Wound healing assay to evaluate wound closure and cell migration ability at different time points (Clone 1).

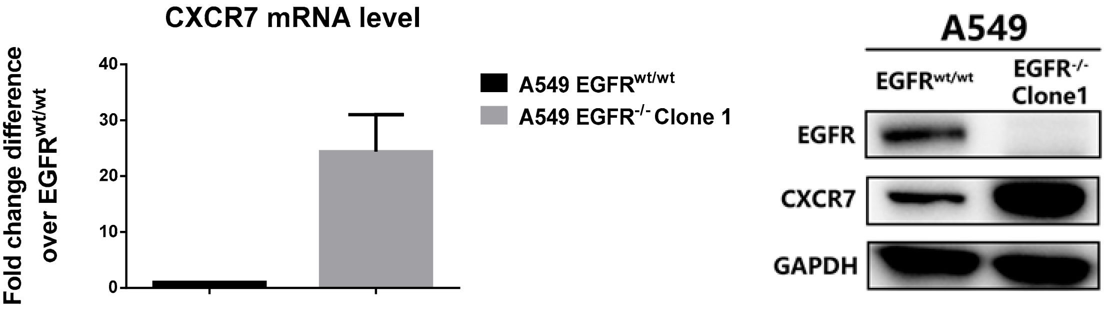

Figure S4. qPCR and Western blot analysis of CXCR7 in A549 *EGFR*$^{wt/wt}$ and *EGFR*$^{-/-}$ cells (Clone 1).

Table S1. List of gRNA sequences for CRISPR/Cas9.

Name	Strand	Sequence
sgRNA-1	F	5′-TGAGCTTGTTACTCGTGCCT-3′
	R	5′-AGGCACGAGTAACAAGCTCA-3′
sgRNA-2	F	5′-GAGTAACAAGCTCACGCAGT-3′
	R	5′-ACTGCGTGAGCTTGTTACTC-3′
sgRNA-3	F	5′-ATAGTTAGATAAGACTGCTA-3′
	R	5′- TAGCAGTCTTATCTAACTAT-3′

Table S2. List of the primer sets used for qRT-PCR.

Name	Strand	Sequence
CXCR7	F	5′-TGGGCTTTGCCGTTCCCTTC-3′
	R	5′-TCTTCCGGCTGCTGTGCTTC-3′
CXCL12	F	5′-GAGCCAACGTCAAGCATCTCA-3′
	R	5′-TTTAGCTTCGGGTCAATGCAC-3′
α-catenin	F	5′-CTCTACTGCCACCAGCTGAACATC-3′
	R	5′-ATGCCTTCACTGTCTGCACCAC-3′
E-Catenin	F	5′-TACACTGCCCAGGAGCCAGA-3′
	R	5′-TGGCACCAGTGTCCGGATTA-3′
N-Catenin	F	5′-TGGGAATCCGACGAATGG-3′
	R	5′-TGCAGATCGGACCGGATACT-3′
Vimentin	F	5′-CCTTGAACGCAAAGTGGAATC-3′
	R	5′-GACATGCTGTTCCTGAATCTGAG-3′
HER2	F	5′-TCTGGACGTGCCAGTGTGAA-3′
	R	5′-CCTGCTCCCTGAGGACACAT-3′
HER3	F	5′-CGGTTATGTCATGCCAGATACAC-3′
	R	5′-GAACTGAGACCCACTGAAGAAAGG-3′
HER4	F	5′-GAGGCTGCTCAGGACCTAAGG-3′
	R	5′-GAGTAACACATGCTCCACTGTCATT-3′
IL-8	F	5′- TGCAGTTTTGCCAAGGAGTG -3′
	R	5′- CAACCCTCTGCACCCAGTTT-3′
